# Age and sex dependent effects of early overnutrition on metabolic parameters and the role of neonatal androgens

**DOI:** 10.1186/s13293-016-0079-5

**Published:** 2016-05-18

**Authors:** Pilar Argente-Arizón, Purificación Ros, Francisca Díaz, Esther Fuente-Martin, David Castro-González, Miguel Ángel Sánchez-Garrido, Vicente Barrios, Manuel Tena-Sempere, Jesús Argente, Julie A. Chowen

**Affiliations:** Department of Endocrinology, Hospital Infantil Universitario Niño Jesús, Instituto de Investigación La Princesa, Avenida Menéndez Pelayo 65, Madrid, 28009 Spain; Department of Pediatrics, Universidad Autónoma of Madrid, Madrid, Spain; Centro de Investigación Biomédica en Red de la Fisiopatología de la Obesidad y Nutrición (CIBEROBN), Instituto de Salud Carlos III, Madrid, Spain; Hospital Universitario Puerta de Hierro-Majadahonda, Madrid, Spain; Department of Cell Biology, Physiology and Immunology, University of Córdoba, Instituto Maimónides de Investigación Biomédica de Córdoba (IMIBIC/HURS), Córdoba, 14004 Spain

**Keywords:** Neonatal nutrition, Metabolic hormones, Sex differences, Litter size, Neonatal testosterone

## Abstract

**Background:**

Males and females respond differently to diverse metabolic situations. Being raised in a small litter is reported to cause overnutrition that increases weight gain and predisposes an individual to metabolic disturbances in adulthood; however, existing data are inconsistent. Indeed, significant weight gain and/or metabolic disturbances, such as hyperinsulinemia and hyperleptinemia, are sometimes not encountered. We hypothesized that these inconsistencies could be due to the animal’s sex and/or age at which metabolic parameters are measured.

**Methods:**

To analyze the effects of neonatal overnutrition, male and female Wistar rats were raised in litters of 4 or 12 pups/dam and killed at postnatal days (PND) 10, 21, 30, 50, 85, or 150. In a second study to determine if neonatal sex steroid levels influence sex differences in metabolic parameters, female rats were treated with testosterone on PND1. Effects on weight, length, fat pads, adipokine production, and serum levels of glucose, metabolic hormones, and cytokines were analyzed in both studies.

**Results:**

By PND10, both males and females raised in small litters had increased body weight, body length, adiposity, and serum glucose, insulin, leptin, and adiponectin levels. Females had a greater increase in inguinal fat, and males had higher expression of leptin messenger RNA (mRNA) and serum insulin, as well as increased testosterone levels. Most of the litter size effects diminished or disappeared after weaning and reappeared during adulthood in males, with sex differences in body size and adiposity being apparent postpubertally. Treatment of females with testosterone on PND1 tended to masculinize some metabolic parameters in adulthood such as increased body weight and serum leptin levels.

**Conclusions:**

Our results indicate that (1) both sex and age determine the response to neonatal overnutrition; (2) differences in neonatal sex steroid levels may participate in the development of sex differences in metabolic parameters in adulthood and possibly in the response to neonatal overnutrition; and (3) the comparison of circulating hormone and cytokine levels, even in normal control animals, should take into consideration the early neonatal nutritional environment.

## Background

Poor nutrition, the lack of sufficient exercise, and how these factors interact with an individual’s underlying genetic make-up clearly contribute to excess body weight gain. However, the current obesity epidemic may also be propelled by the fact that new generations are being exposed to factors during early development that can increase their propensity to become overweight or obese [1, 2], even if they ingest a normal diet throughout postnatal life, as observed in animal studies [3–8]. In animal studies, poor maternal nutrition and/or health and fetal/neonatal exposure to an inadequate diet, stress, abnormal levels of hormones, and environmental toxins have all been shown to disrupt metabolic homeostasis [3–10], with some of these effects also being reported in humans [1, 2, 11, 12]. Some metabolic responses to these early environmental perturbations can differ between male and female rodents [13–18] and may also be age dependent, sometimes only appearing in later adulthood [19–21]. However, the extent to which males and females have different metabolic responses to early nutritional changes, and whether these differential responses are age dependent, remains to be thoroughly studied.

In experimental animal models, modification of the litter size in which animals are raised can have long-term effects on their metabolic homeostasis, with small litter rearing promoting overfeeding and large litter rearing resulting in under-nutrition [13, 22–24]. These long-term effects are suggested to be due to changes in food intake during nursing and/or maternal nurturing [25, 26]. Although some long-term outcomes of this relatively non-invasive experimental manipulation have been shown to be sex specific [13, 18, 27, 28], the majority of studies using this paradigm were not performed simultaneously in males and females. Hence, our aim was to compare the metabolic responses of male and female rats to neonatal overnutrition throughout development. Moreover, as increased adiposity is often associated with a rise in circulating cytokines, at least in adults [29–31], we asked whether early overnutrition and increased adiposity results in changes in circulating cytokine levels. Postpubertal differences in body size and composition are at least partially due to gonadal sex steroids [32, 33]. However, sex differences in hormone levels during early stages of development, such as the testosterone surge observed in neonatal male rodents [34], could also be involved in determining long-term metabolic outcomes. Hence, our aim was to evaluate the effect of litter size on changes in body weight, adiposity, and circulating metabolic hormones and cytokines, as well as adipokine and cytokine production by different adipose depots, at different stages of postnatal development in rats of both sexes. In addition, we analyzed whether a reduction in litter size modifies the neonatal sex steroid environment and if experimental modification of testosterone levels in female rats has long-term effects on metabolic parameters. We report different outcomes between males and females in response to neonatal overnutrition as early as postnatal day (PND) 10, with some of these effects possibly being due to increased circulating testosterone levels in male rats exposed to neonatal overnutrition.

## Methods

### Animals

All experiments were approved by the University of Córdoba Ethical Committee for Animal Experimentation and the Research Commission of the Hospital Infantil Universitario Niño Jesús and complied with the Royal Decree 53/2013 and with the European Union guidelines for use of experimental animals (2010/63/EU).

Adult Wistar rats were purchased from Harlan Interfauna Ibérica S.A. (Barcelona, Spain) and allowed to acclimate for 2 weeks before mating. For mating, one male was placed in a cage with three virgin females (all weighing approximately 150 g) for 2 days. Each female was then housed separately. Rats were maintained at a constant temperature (21 ± 1 °C) and humidity (50 ± 1 %) in a 12-h light–dark cycle (lights on at 07:30) and given free access to rat chow (A04-10/15022 Panlab, Barcelona, Spain) and water.

### Neonatal overnutrition: litter organization

Only mothers that gave birth to between 8 and 12 pups were employed for the study (mean 10.3 ± 0.2 pups born/litter). On the day of birth, PND0, pups were organized into litters of 4 (L4: 2 males and 2 females) or 12 pups (L12: 6 males and 6 females), employing cross-fostering such that pups from at least three different dams that gave birth within 12 h of each other were used to form each new litter. A total of 12 litters of 12 pups and 36 litters of 4 pups were employed in these studies. The litters were arranged such that the mean birth weights did not differ between groups (males L4: 5.8 ± 0.1 g, L12: 6.0 ± 0.1 g; females L4: 5.7 ± 0.1 g, L12: 5.7 ± 0.1 g).

One group of pups was killed on PND10 and another on PND21. All remaining pups were removed from their mother on PND21 and placed two per cage according to sex and litter size. All rats were allowed free access to food (normal rat chow) and water and sacrificed at PNDs 30, 50, 85, or 150. These sacrifices were arranged such that six different litters were represented at each time-point for each experimental group. For the PND10 time-point, entire litters were sacrificed. There was a minimum of six rats in all experimental groups at each age analyzed for each parameter.

All rats were weighed and measured (from tip of the nose to the base of the tail) weekly starting at PND21 until they were killed. Food intake was measured weekly by placing a known amount of food in the cage and weighing the remaining amount of food at the same time the following day. To account for spillage, food was retrieved from the bedding before weighing. The number of cages was used as the *n* for statistical analysis.

Before being killed by decapitation and being fasted overnight, all rats were weighed and their length measured. Glycemia was analyzed (Optimum Xceed, Abbot) in a drop of blood from the tip of the tail. On the day prior to being killed, a vaginal swab was taken on all postpubertal females to determine the estrous stage. Approximately 80 % were in estrous and 20 % diestrous, with no difference between groups. Inguinal and perigonadal fat pads were rapidly removed, weighed and frozen in dry ice. Trunk blood was collected, allowed to clot, centrifuged (3000 rpm during 10 min at 4 °C) and the serum removed. All tissues were stored at −80 °C until processed.

### Neonatal androgen treatment

For this study three groups of rats were generated, males (M), females (F) and androgenized females (AF), and sacrificed at PND10 and PND85-90, two ages where we had observed differences between the sexes in specific parameters in the previous experiment. On the day of birth, these rats were arranged in litters of 10 rats/dam (5 males and 5 females), in order to represent a normal litter size. On PND1, female rats received a subcutaneous injection of 1.25 mg of testosterone propionate (approximately 208 mg/kg, Sigma) dissolved in 100 μL of olive oil. Control females and males received 100 μL of olive oil.

Half of each group was killed on PND10 (10 rats/group) and half on approximately PND90 (*N* = 9 for males, *N* = 12 for females and *N* = 11 for androgenized females). Androgenized females do not display vaginal opening and are acyclic. In control females, the stage of the estrous cycle was monitored by performing vaginal swabs and they were sacrificed between PNDs 85–90 on the morning of diestrous. All PND90 animals were fasted for 12 h before sacrifice. Body weight, length and glycemia were measured on the day of sacrifice. Inguinal and perigonadal fat pads were rapidly removed, weighed, and frozen on dry ice. Trunk blood was collected, allowed to clot, centrifuged (3000 rpm during 10 min at 4 °C) and the serum removed. All tissues were stored at −80 °C until processed.

### Serum hormone analysis

Circulating leptin, insulin, interleukin (IL) 1β, IL6, and tumor necrosis factor α (TNFα) were determined by multiplexed immunoassay according to the manufacturer’s instructions (Millipore, Billerica, MA) in a Bio-Plex suspension array system 200 (Bio-Rad Laboratories, Hercules, CA, USA). Mean fluorescence intensity was analyzed by using Bio-Plex Manager Software 4.1.

Serum adiponectin (Millipore, Billerica, MA, USA), testosterone (Cusabio, Wuhan, PR China), and 17β-estradiol (Cusabio) concentrations were determined by ELISA as indicated by the manufacturer.

All samples were run in duplicate and within the same assay for all analyses. The limit of detection and the intra- and inter-coefficients of variation for all analyses are shown in Table 1.

**Table 1:** The limit of detection and intra- and inter-assay coefficients (CV) of variation for the hormone and cytokine assays

	Detection limit (pg/ml)	CV intra-assay (%)	CV inter-assay (%)
Leptin	21.5	8.2	8.5
Insulin	51	7.3	9.8
Adiponectin	400	1.8	7.3
Testosterone	60	7.7	8.0
Estradiol	<25	11.4	13.2
IL 6	9.8	10.4	14.2
IL1β	2.4	7.6	12.4
TNFα	4.5	9.2	11.1

### RNA isolation and real-time PCR

Total mRNA was isolated from adipose tissue by using TRIzol® Reagent (Invitrogen). Complementary DNA (cDNA) was synthesized from 2 μg of total mRNA by using a high capacity cDNA reverse transcription kit (Applied Biosystems, Foster City, CA, USA). Assay-on-demand kits (Applied Biosystems) were used to assess the mRNA levels of leptin (Rn00565158_m1), adiponectin (Rn00595250_m1), IL6 (Rn01410330_m1), IL1β (Rn01336189_m1), and TNFα (Rn01525859_g1) quantitative real-time PCR according to the manufacturer’s protocol and analyzed in an ABI PRISM 7000 Sequence Detection System (Applied Biosystems).

Various housekeeping genes were analyzed to find a stable internal control. However, we were unable to find suitable housekeeping genes that did not vary with age, sex and litter size, as previously reported by others [35]. Thus, the mRNA levels of the genes analyzed in adipose tissue were not compared between ages. Results are compared at each age to determine the effects of sex and litter size and were normalized to two of the following: phosphoglycerate kinase 1 (Pgk-1, Rn00821429_g1), cyclophilin A (PPIA, Rn00690933_m1), or ribosomal protein S 18-like (Rps18, Rn01428915_g1). The housekeeping genes used did not change between groups at a given age. The ΔΔCT method was used to determine relative expression levels and this was used for statistical analysis. All data are expressed as the percent of the values of L12 males at each age.

### Statistical analysis

The program STATVIEW, version 5.0 (SAS Institute Inc., Cary, NC, USA) was used for data analysis. To rule-out an effect of the litter in which rats were reared (dam effect), analyses using rearing litter as a factor, as well as a cofactor, were performed within each litter size (i.e., 4 or 12 pups). No effect was found on any parameter analyzed. Three-way ANOVA was used to determine the effect and interaction of litter size, sex, and age on the variables analyzed. When significant effects were found, this was followed by two-way (split by litter size, sex or age) and one-way ANOVAs when and where appropriate. When found to be statistically significant, the one-way ANOVA was then followed by Scheffe’s f test for post hoc analyses. To determine differences in weight, length, and food intake over time, the analyses included repeated measures. In the case of mRNA levels in adipose tissue, where no comparison between ages was possible, two-way ANOVAs (factors of litter size and sex) were first performed, followed by one-way ANOVAs and Scheffe’s *f* test when appropriate. The results were considered statistically significant at *p* < 0.05. All data are presented as mean ± SEM. The *p* values reported in the figures correspond to the one-way ANOVA and the comparisons on the graphs and in the tables represent the results of the post hoc analyses. Only physiologically significant differences (i.e., with only one factor (sex, age or litter size) being different between the groups of comparison) are represented.

## Results

### Effect of litter size and sex on body weight, length, and food intake throughout development

#### Body weight

At birth, males weighed more than females (males 6.00 ± 0.04 g, females 5.72 ± 0.04 g; *F*_(1,284)_ = 34.1, *p* < 0.0001). The litters were cross-fostered such that the mean starting weight of each sex did not differ between groups.

**Fig. 1:**
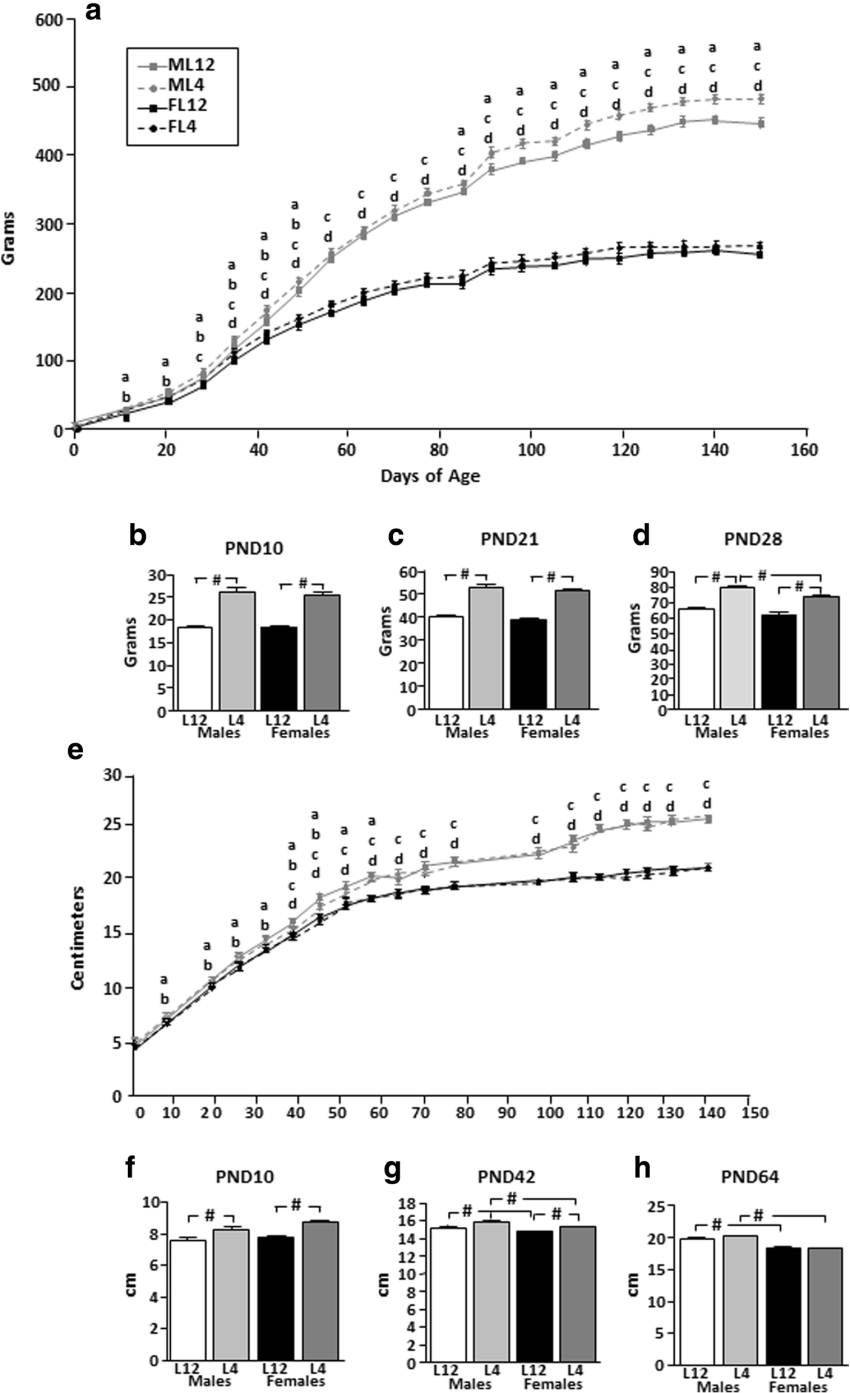
**a** Body weight gain over time in male (M) and female (F) rats reared in litters of 4 (L4) or 12 (L12) pups. Magnification of the differences in body weight at **b** postnatal day (PND) 10, **c** PND21, and **d** PND28. **e** Body length over time and magnification of the differences in length at **f** PND10, **g** PND42, and **h** PND64. *a* = M4 different from M12, *b* = F4 different from F12, *c* = M4 different from F4, *d* = M12 different from F12. #*p* < 0.0001. *N* = a minimum of 12 for each experimental group and age

There was an effect of sex (*F*_(1,33)_ = 327.9, *p* < 0.0001), litter size (*F*_(1,33)_ = 6.6, *p* < 0.02), and age (*F*_(19,627)_ = 3245.3, *p* < 0.0001) on body weight throughout the study (Fig. 1a), with interactions between sex and age (*F*_(19,627)_ = 335.5, *p* < 0.0001) and litter size and age (*F*_(19,627)_ = 1.9, *p* < 0.05).

The body weights of males and females were not significantly different until approximately PND28, when L4 males were found to weigh more than L4 females (Fig. 1a-d; sex effect *F*_(1,170)_ = 9.1, *p* < 0.003). Throughout the rest of the study, males weighed more than females regardless of their experimental group.

As early as PND10, L4 rats weighed more than those from L12 (litter size effect: *F*_(1,44)_ = 125.2, *p* < 0.0001; Fig. 1b) with this effect persisting at PND21 (litter size effect: *F*_(1,234)_ = 212.7, *p* < 0.0001; Fig. 1c) and PND28 (litter size effect: *F*_(1,170)_ = 73.5, *p* < 0.0001). The influence of litter size on body weight continued until approximately PND60, at which time it was no longer observed in either sex. This effect was again statistically significant from approximately PND90 onward in males (ML12: 379.5 ± 9.5, ML4: 417.0 ± 5.2, FL12: 235.9 ± 5.4, FL4: 244.8 ± 6.7 g; F_(3,44)_ = 148.8, *p* < 0.0001). Male rats from L4 weighed more than those from L12 throughout the rest of the study.

#### Body length

At birth, males were longer than females (sex effect: *F*_(1,246)_ = 27.3, *p* < 0.0001; ML12: 4.82 ± 0.03, ML4: 4.83 ± 0.02, FL12: 4.68 ± 0.03, FL4: 4.70 ± 0.03 cm).

Throughout the study, body length (Fig. 1e) was determined by sex (*F*_(1,410)_ = 270.0, *p* < 0.0001) and age (*F*_(10,410)_ = 4815.8, *p* < 0.0001), with interactions between these two factors (*F*_(10,410)_ = 35.7, *p* < 0.0001), as well as litter size and age (*F*_(10,410)_ = 8.7, *p* < 0.0001).

At PND10, there was no difference between sexes (Fig. 1f). Males began to be significantly longer than females around PND42 (Fig. 1g), which is coincident with the normal period of pubertal development of male rats. Males were longer than females at all subsequent ages.

At PND10, L4 rats of both sexes were longer than L12 rats (Fig. 1f). This effect of litter size remained until approximately PND64 (Fig. 1h).

#### Mean food intake per day throughout the study

**Fig. 2:**
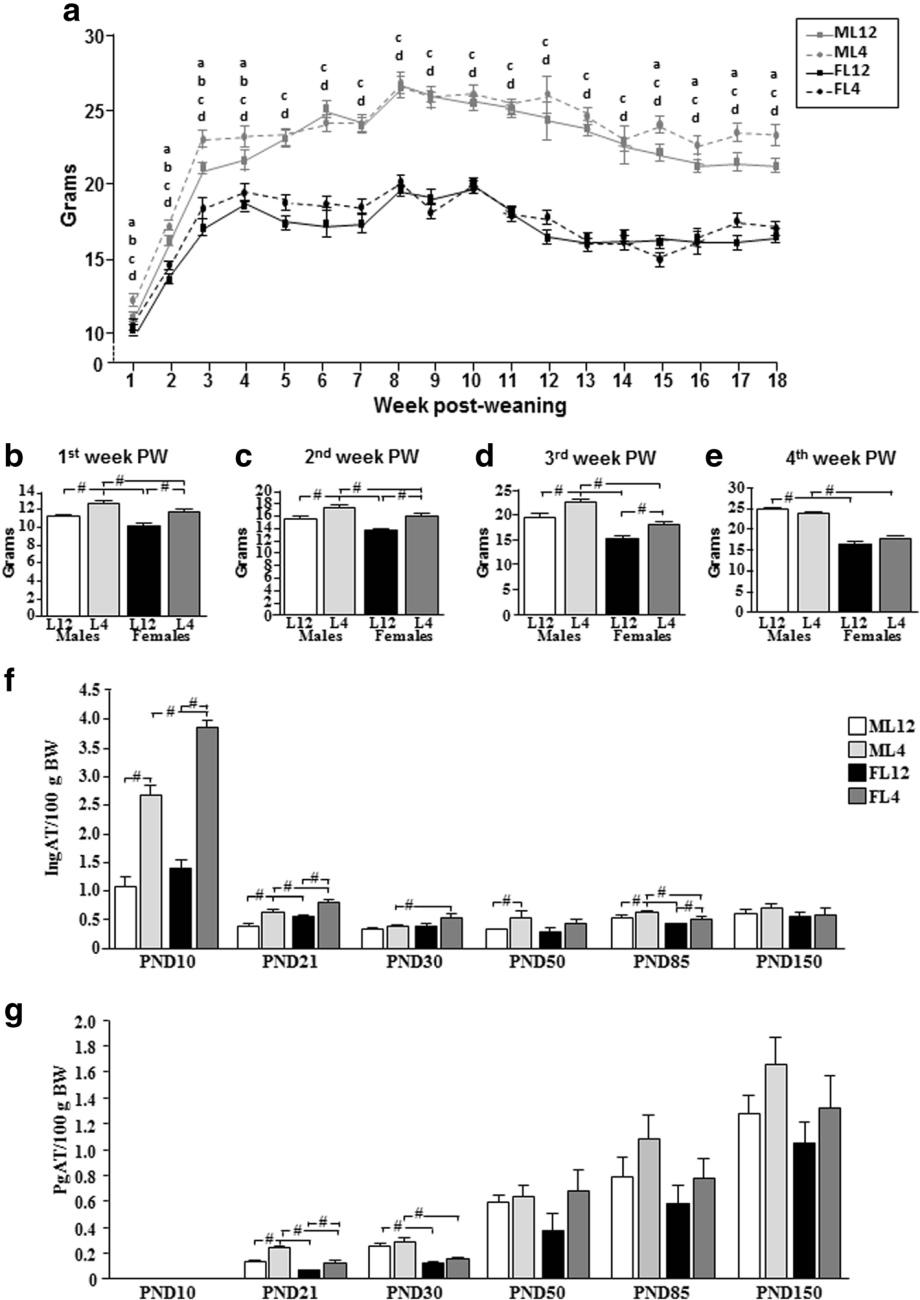
**a** Mean daily food intake in male (M) and female (F) rats reared in litters of 4 (L4) or 12 (L12) pups from the time they were weaned until the end of the study at postnatal day (PND) 150. The mean daily food intake during the **b** first, **c** second, **d** third, and **e** fourth weeks postweaning (PW). **f** The mean percentage of inguinal adipose tissue (IngAT) starting at PND10 and **g** perigonadal adipose tissue (PgAT) starting at PND21 in rats throughout development. At PND10, PgAT was unappreciable. #*p* < 0.0001. *a* = M4 different from M12, *b* = F4 different from F12, *c* = M4 different from F4, *d* = M12 different from F12. #*p* < 0.0001. *BW* body weight. *N* = a minimum of six for each experimental group and at each age

Food intake was affected by sex over time (*F*_(8,272)_ = 25.2, *p* < 0.0001; Fig. 2a). Males ate more than females as early as the first week after weaning (sex effect: *F*_(1,120)_ = 13.6, *p* < 0.0003) and throughout the study.

Male and female rats from L4 ate more than those from L12 (litter size effect: *F*_(1,120)_ = 27.6, *p* < 0.0001) during the first, (Fig. 2b), second (Fig. 2c) and third (Fig. 2d) weeks postweaning. During the fourth week postweaning, the litter size effect on food intake was no longer observed in either sex (Fig. 2e) and this remained so until approximately PND110 when L4 males began to again eat significantly more than L12 rats.

The overall mean daily food intake from weaning until the end of the study was greater in males than in females, regardless of litter size (ML12: 21.7 ± 0.3, ML4: 21.9 ± 0.4, FL12: 16.5 ± 0.2, FL4: 17.0 ± 0.2 g/rat/day; *p* < 0.001). However, although litter size had an effect at specific time-points throughout development, the overall mean daily food intake throughout the study was not affected by litter size in either sex.

#### Effect of litter size and sex on body fat content and distribution

Although there was no effect of litter size on body weight in early adulthood, we hypothesized that body composition might differ. Moreover, males and females are reported to differ in the distribution of body fat, at least postpubertally [36–38]. Hence, we analyzed the weights of the inguinal and perigonadal adipose depots at different postnatal ages.

#### Inguinal adipose tissue

There was an effect of sex (*F*_(1,164)_ = 96.9, *p* < 0.0001), litter size (*F*_(1,164)_ = 53.9, *p* < 0.0001), and age (*F*_(5,164)_ = 179.9, *p* < 0.0001) on absolute inguinal fat mass, with interactions between sex and age (*F*_(5,164)_ = 44.4, *p* < 0.0001), litter size and age (*F*_(5,164)_ = 2.53, *p* < 0.04) and sex, litter size, and age (*F*_(4,164)_ = 2.6, *p* < 0.03; data not shown).

When the amount of inguinal adipose tissue was normalized to body weight (g/100 g BW), these effects remained [sex (*F*_(1,164)_ = 4.9, *p* < 0.03), litter size (*F*_(1,164)_ = 92.2, *p* < 0.0001), and age (*F*_(5,164)_ = 211.5, *p* < 0.0001)] with interactions between sex and age (*F*_(5,164)_ = 11.1, *p* < 0.0001), litter size and age (*F*_(5,164)_ = 62.7, *p* < 0.0001) and sex, litter size, and age (*F*_(5,164)_ = 3.0, *p* < 0.003).

The relative amount of inguinal adipose tissue was highest at PND10 compared to all latter ages (Fig. 2f). At this early age, there was an effect of sex (*F*_(1,46)_ = 19.8, *p* < 0.0001), with an interaction between sex and litter size (*F*_(1,46)_ = 6.7, *p* < 0.01), as L4 females had higher levels compared to L4 males. At weaning (PND21), females had relatively more inguinal adipose tissue than males regardless of litter size (sex effect: *F*_(1,45)_ = 19.8, *p* < 0.0001). At PND30, L4 females continued to have more inguinal adipose tissue than L4 males (*F*_(1,16)_ = 4.8, *p* < 0.05). At PND85, there continued to be an effect of sex (*F*_(1,20)_ = 8.6, *p* < 0.01), but at this age males had more inguinal adipose tissue than females regardless of litter size, with this overall effect of sex continuing at PND150 (*F*_(1,19)_ = 5.2, *p* < 0.05).

As early as PND10, rats from L4 had relatively more inguinal adipose tissue than those from L12 (litter effect: *F*_(1,46)_ = 151.3, *p* < 0.0001). When weaned (PND21), L4 rats continued to have more inguinal adipose tissue than L12 rats (*F*_(1,45)_ = 54.1, *p* < 0.0001). No effect of litter size was found at PND30, but at PND50 (litter effect: *F*_(1,20)_ = 6.5, *p* < 0.02) and PND85 (litter effect: *F*_(1,20)_ = 4.5, *p* < 0.05) L4 rats had more inguinal adipose tissue than L12 rats. This effect was no longer seen at PND150.

#### Perigonadal adipose tissue

At PND10 there was insufficient perigonadal adipose to be analyzed. Throughout the remaining ages, the weight of the perigonadal adipose tissue depot was affected by sex (*F*_(1,118)_ = 46.1, *p* < 0.0001) and age (*F*_(4,118)_ = 146.4, *p* < 0.0001) with an interaction between sex, litter size and age (*F*_(4,118)_ = 3.0, *p* < 0.05). When perigonadal adipose mass was normalized to body weight there was an effect of sex (*F*_(1,118)_ = 5.2, *p* < 0.05), litter size (*F*_(1,118)_ = 4.1, *p* < 0.05) and age (*F*_(4,118)_ = 137.1, *p* < 0.0001; Fig. 2g).

Males had a higher percentage of perigonadal adipose tissue than females at PND21 (sex effect: *F*_(1,39)_ = 37.3, *p* < 0.0001) and PND30 (sex effect: *F*_(1,18)_ = 40.6, *p* < 0.0001). Postpubertally males tended to have more perigonadal adipose tissue than females and L4 rats more than L12 rats, but these differences were not significant most likely due to the high variability in the data.

At PND21, L4 rats of both sexes had a higher percentage of perigonadal adipose tissue than L12 rats (*F*_(1,39)_ = 42.8, *p* < 0.0001). No effect of litter size was found at any of the latter ages.

### Effect of litter size and sex on circulating hormones and cytokines

Glucose levels (Table 2) were affected by litter size (*F*_(1,248)_ = 10.4, *p* < 0.002) and age (*F*_(5,248)_ = 139.4, *p* < 0.0001) with an interaction between these two factors (*F*_(5,248)_ = 6.2_,_*p* < 0.0001). At PND10 (sex effect: *F*_(1,44)_ = 3.7, *p* < 0.2) and PND21 (sex effect: *F*_(1,42)_ = 9.3, *p* < 0.0001), L4 rats had significantly higher glucose levels than L12 rats regardless of sex. At PND30, glycemia decreased in rats of all groups. Between PND85 and PND150 glycemia rose significantly in all groups except female L12 rats, where this rise was more gradual starting at PND50.

**Table 2:** Glycemia and serum hormone and cytokine levels at postnatal days (PND) 10, 21, 30, 50, 85, and 150 in male (M) and female (F) rats raised in litters (L) 4 or 12 pups

	Glucose (mg/dl)	Insulin (ng/ml)	HOMA	Leptin (ng/ml)	Adiponectin (ng/ml)	Il1β (pg/ml)	IL6 (pg/ml)	TNFα (pg/ml)
ML12PND10^d^	114.4 ± 4.1	0.44 ± 0.07	3.2 ± 0.5	1.25 ± 0.21	21.5 ± 2.8	14.9 ± 3.1	126.1 ± 38.5	5.1 ± 0.3
ML4PND10^d^	130.9 ± 5.9^a^	0.92 ± 0.10^a^	7.7 ± 1.2^a^	2.61 ± 0.32^a^	40.0 ± 2.3^a^	5.1 ± 1.2	89.2 ± 44.6	3.4 ± 0.7^a^
FL12PND10^d^	121.6 ± 5.1	0.34 ± 0.02	2.4 ± 0.2	1.24 ± 0.16	17.5 ± 2.3	15.0 ± 4.1	16.4 ± 12.2	4.0 ± 0.6
FL4PND10^d^	135.8 ± 4.5^a^	0.60 ± 0.06 ^a, b^	4.8 ± 0.5 ^a, b^	2.40 ± 0.32^a^	41.9 ± 1.5^a^	7.9 ± 2.0	40.9 ± 29.7	1.3 ± 0.4^a, b^
ML12PND21^d^	103.6 ± 5.3	0.32 ± 0.05	2.1 ± 0.4	1.12 ± 0.18	27.6 ± 1.8	19.6 ± 6.8	56.5 ± 44.6	4.6 ± 1.0
ML4PND21^d^	131.7 ± 5.5 ^a^	0.69 ± 0.24	5.4 ± 1.9^a^	1.80 ± 0.21^a^	31.9 ± 1.6	46.9 ± 11.3	85.6 ± 39.7	2.5 ± 1.2
FL12PND21^d^	109.3 ± 2.9	0.22 ± 0.04	1.5 ± 0.3	0.99 ± 0.22	30.3 ± 1.7	28.0 ± 9.4	60.4 ± 34.3	2.5 ± 0.8
FL4PND21^d^	125.6 ± 3.1 ^a^	0.80 ± 0.16	6.0 ± 1.3^a^	1.91 ± 0.48^a^	43.9 ± 3.2 ^a, b^	22.8 ± 3.7	112.1 ± 69.8	1.9 ± 0.6
ML12PND30	68.2 ± 2.9^c^	0.19 ± 0.03	0.8 ± 0.1^c^	0.43 ± 0.12	62.1 ± 6.0^c^	32.1 ± 8.0	76.2 ± 47.4	1.6 ± 0.4
ML4PND30	76.9 ± 4.7 ^c^	0.23 ± 0.05	1.1 ± 0.2^c^	0.97 ± 0.44	58.4 ± 6.3^c^	61.3 ± 41.4	18.4 ± 14.4	2.0 ± 0.9
FL12PND30	72.7 ± 4.8 ^c^	0.13 ± 0.01	0.6 ± 0.1^c^	0.71 ± 0.32	42.6 ± 3.0^b^	67.5 ± 29.0	86.5 ± 35.0	4.1 ± 1.3
FL4PND30	75.8 ± 4.0 ^c^	0.57 ± 0.37	2.6 ± 1.8^c^	1.04 ± 0.54	54.2 ± 5.7	29.2 ± 13.6	78.3 ± 23.8	2.1 ± 0.7
ML12PND50	65.7 ± 3.3	0.89 ± 0.44	3.5 ± 1.8	0.96 ± 0.32	55.2 ± 4.2	10.6 ± 2.3	18.1 ± 17.5	BLD
ML4PND50	63.8 ± 3.8	0.73 ± 0.36	3.1 ± 1.7	0.69 ± 0.29	76.2 ± 5.9 ^a^	20.9 ± 3.9	0.62	BLD
FL12PND50	63.8 ± 2.4	0.71 ± 0.31	2.6 ± 1.1	0.77 ± 0.29	62.2 ± 10.1	19.3 ± 8.9	17.7 ± 12.7	BLD
FL4PND50	69.7 ± 3.4	0.37 ± 0.07	1.6 ± 0.3	0.69 ± 0.31	60.9 ± 9.7	7.3 ± 2.4	22.1 ± 11.4	BLD
ML12PND85	68.8 ± 3.7	0.70 ± 0.18	3.1 ± 0.9	1.65 ± 0.20	71.6 ± 14.0	15.6 ± 5.5	5.6 ± 5.0	BLD
ML4PND85	61.0 ± 2.4	0.94 ± 0.37	3.5 ± 1.3	1.42 ± 0.21	78.0 ± 19.1	15.7 ± 6.6	13.5 ± 12.9	BLD
FL12PND85	73.3 ± 3.4	0.15 ± 0.03	0.7 ± 0.2	1.22 ± 0.44	108.2 ± 9.1 ^b^	6.1 ± 2.1	4.6 ± 3.9	BLD
FL4PND85	66.3 ± 2.9	0.60 ± 0.22	2.2 ± 0.9	1.04 ± 0.18	105.2 ± 2.5 ^b^	6.7 ± 2.1	BLD	BLD
ML12PND150	85.7 ± 4.0^c^	3.46 ± 0.99^c^	13.5 ± 4.4^c^	3.89 ± 0.82^c^	87.0 ± 9.0	23.4 ± 9.6	14.5 ± 6.2	BLD
ML4PND150	78.4 ± 4.0^c^	2.82 ± 0.25^c^	12.1 ± 1.1 ^c^	3.60 ± 0.88^c^	108.8 ± 7.3	35.3 ± 20.0	15.5 ± 3.6	BLD
FL12PND150	81.3 ± 8.7	1.03 ± 0.35^c^	3.8 ± 1.4^b, c^	2.32 ± 0.20^b, c^	106.1 ± 7.7	29.3 ± 26.4	16.4 ± 3.7	BLD
FL4PND150	84.9 ± 6.3^c^	1.55 ± 0.40^c^	6.1 ± 1.7^b,c^	2.57 ± 0.37^b, c^	86.4 ± 12.4	22.6 ± 8.0	19.1 ± 0.7	BLD

*N* = 6–8 in each experimental group at each age

*HOMA* homeostatic model assessment, *BLD* below limit of detection

^a^Different from rats of same age and sex (litter size effect)

^b^Different from males of same age and litter size

^c^Different from preceding age (same sex and litter size)

^d^Non-fasting samples

Insulin levels (Table 2) were affected by age (*F*_(5,149)_ = 24.4, *p* < 0.0001) and sex (*F*_(1,149)_ = 13.0, *p* < 0.0001), with an interaction between age and sex (*F*_(5,149)_ = 6.4, *p* < 0.0001). At PND10, there was an effect of sex (*F*_(1,20)_ = 9.5, *p* < 0.006), with L4 males having higher levels than L4 females (*F*_(3,20)_ = 13.9, *p* < 0.0001). After PND10, there were no differences between male and female insulin levels until PND150 (sex effect: *F*_(1,22)_ = 4.7, *p* < 0.05), when males had higher levels than females. L4 rats had higher insulin levels than L12 rats at PND10 (litter effect: *F*_(1,20)_ = 29.5, *p* < 0.0001) and PND21 (litter effect: *F*_(1,20)_ = 10.0, *p* < 0.005). After PND21, no litter effect was found in either sex. Insulin increased significantly at PND150 in all groups.

As an index of insulin sensitivity, homeostatic model assessment (HOMA) was calculated [insulin (mIU/ml) × glucose mg/dl/405]. There was an effect of sex (*F*_(1,132)_ = 13.0, *p* < 0.0005), litter size (F_(1,132)_ =7.4, *p* < 0.008), and age (*F*_(5,132)_ =16.3, *p* < 0.0001), with an interaction between sex and age (*F*_(5,132)_ = 5.4, *p* < 0.0002). Males had a higher HOMA than females at PND10 (sex effect: *F*_(1,20)_ = 6.5, *p* < 0.02) and PND150 (*F*_(1,22)_ = 4.8, *p* < 0.01). At PND10, L4 rats of both sexes had a higher HOMA index than L12 animals (litter effect: *F*_(1,20)_ = 23.7, *p* < 0.0001) , with L4 males having a higher index than L4 females (*F*_(3,20)_ = 10.9, *p* < 0.0002). The effect of litter size was maintained in both sexes at PND21 (*F*_(1,20)_ = 10.9, *p* < 0.004). There were no differences between groups at PND30, 50, or 85. In females, HOMA decreased between PND21 and PND30 and then rose again at PND150 regardless of litter size (*F*_(11,65)_ = 3.02, *p* < 0.003). In males, HOMA decreased between PND21 and PND30 and then increased between PND85 and PND150 (*F*_(11,68)_ = 7.0, *p* < 0.0001).

Circulating leptin levels (Table 2) were dependent on sex (*F*_(1,129)_ = 3.7, *p* < 0.05), age (*F*_(5,129)_ = 21.1, *p* < 0.0001), and litter size (*F*_(1,129)_ = 4.9, *p* < 0.3), with an interaction between the latter two factors (*F*_(5,129)_ = 3.0, *p* < 0.02). Males had higher leptin levels than females only at PND150 (sex effect: *F*_(1,22)_ = 5.7, *p* < 0.5). At PND10 (litter effect: *F*_(1,20)_ = 9.9, *p* < 0.005) and PND21 (litter effect: *F*_(1,18)_ = 9.3, *p* < 0.007), L4 rats had higher levels than L12 rats. Serum leptin increased in all groups between PND85 and PND150.

There was an effect of litter size (*F*_(1,113)_ = 7.1, *p* < 0.009) and age (*F*_(5,113)_ = 61.8, *p* < 0.0001) on circulating adiponectin levels (Table 2), with an interaction between sex and age (*F*_(5,113)_ = 4.2, *p* < 0.002) and sex, litter size, and age (*F*_(5,113)_ = 2.4, *p* < 0.05). Adiponectin levels increased with age. At PND10, L4 rats had higher levels than L12 rats in both sexes (litter effect: *F*_(1,20)_ = 108.4, *p* < 0.0001). At PND21, there was an effect of sex (*F*_(1,20)_ = 11.9, *p* < 0.003) and litter size (*F*_(1,20)_ = 16.2, *p* < 0.0007), with an interaction between these two factors (*F*_(1,20)_ = 4.4, *p* < 0.05). At this age, female L4 rats had higher adiponectin levels than L12 females, with no effect found in males and with L4 females having higher levels than L4 males (*F*_(3,20)_ = 10.8, *p* < 0.0002). At PND30, L12 females had lower levels than L12 males (sex effect: *F*_(1,20)_ = 4.8, *p* < 0.05). At PND50, there were no difference between groups, while at PND85 females had higher levels than their corresponding male groups (sex effect: *F*_(1,16)_ = 6.2, *p* < 0.03). At PND150, there was an interaction between sex and litter size (*F*_(1,19)_ = 5.2, *p* < 0.04) with female L12 rats tending to have higher levels than L12 males, with the inverse occurring in L4 rats.

Serum IL1β levels (Table 2) were affected by age (*F*_(5,125)_ = 4.4, *p* < 0.001) with an interaction between sex and litter size (*F*_(1,125)_ = 6.2, *p* < 0.02). When split by age, at PND10, L12 rats of both sexes tended to have higher levels than L4 rats (litter effect: *F*_(1,31)_ = 7.5, *p* < 0.02), but this did not reach significance between individual groups with the post hoc test. There was no litter size effect thereafter. At PND85, the effect of sex approached significance (*F*_(1,20)_ = 4.2; *p* = 0.055), with females having lower levels than males.

Circulating IL6 levels were not significantly affected by any of the experimental factors (Table 2).

At PND10, TNFα levels were affected by sex (*F*_(1,22)_ = 8.9, *p* < 0.008) and litter size (*F*_(1,22)_ = 17.0, *p* < 0.0005), with L4 males having higher levels than L4 females and L12 rats higher than L4 rats (Table 2). At PND21, an overall effect of litter size remained (*F*_(1,19)_ = 6.46, *p* < 0.02). There was no effect of either sex or litter size at PND30. Serum TNFα levels were below the level of detectability of the assay from PND50 onward.

Testosterone levels in males increased with age (ML12PND10: 0.60 ± 0.30, ML4PND10: 3.50 ± 1.14, ML12PND21: 0.60 ± 0.20, ML4PND21: 0.63 ± 0.21, ML12PND30: 0.48 ± 0.24, ML4PND30: 0.35 ± 0.13, ML12PND50: 0.72 ± 0.41, ML4PND50: 0.63 ± 0.15, ML12PND85: 2.60 ± 0.91, ML4PND85: 2.61 ± 0.87, ML12PND150: 11.55 ± 3.42, ML4PND150: 18.68 ± 4.46 ng/ml; age effect: *F*_(5,50)_ = 15.1, *p* < 0.0001). To analyze whether sex differences in testosterone levels occur at PND10, testosterone levels were also measured in females at this age (FL12PND10: 0.34 ± 0.03, FL4PND10: 0.33 ± 0.06). At PND10, there was an effect of litter size (*F*_(1,21)_ = 9.3, *p* < 0.005), sex (*F*_(1,21)_ = 13.1, *p* < 0.002) and an interaction between sex and litter size (*F*_(1,21)_ = 9.5, *p* < 0.005), as L4 males had higher levels than L12 males and L4 females. Testosterone levels did not differ between L4 and L12 males at any other age.

Estradiol levels in females also increased with age (FL12PND10: 11.0 ± 1.5, FL4PND10: 8.5 ± 3.5, FL12PND21: 7.1 ± 3.1, FL4PND21: 17.6 ± 2.2, FL12PND30: 16.7 ± 1.7, FL4PND30: 26.2 ± 3.9, FL12PND50: 22.9 ± 1.6, FL4PND50: 24.1 ± 1.4, FL12PND85: 26.6 ± 4.7, FL4PND85: 24.3 ± 2.0, FL12PND150: 32.5 ± 4.4, FL4PND150: 89.4 ± 18.8 pg/ml; F_(5,52)_ = 42.6, *p* < 0.0001), with an interaction between age and litter size (*F*_(5,52)_ = 2.6, *p* < 0.04). At PND150, L4 rats had higher levels than L12 rats (litter size effect: *F*_(1,16)_ = 7.78, *p* < 0.02).

### Effect of litter size and sex on adipokine expression in adipose tissue

As the percentage of body fat was affected by sex and litter size during early postnatal life and in late adulthood, we analyzed the expression of metabolic and inflammatory related cytokines to determine how adipose tissue is affected.

#### Inguinal adipose tissue

**Fig. 3:**
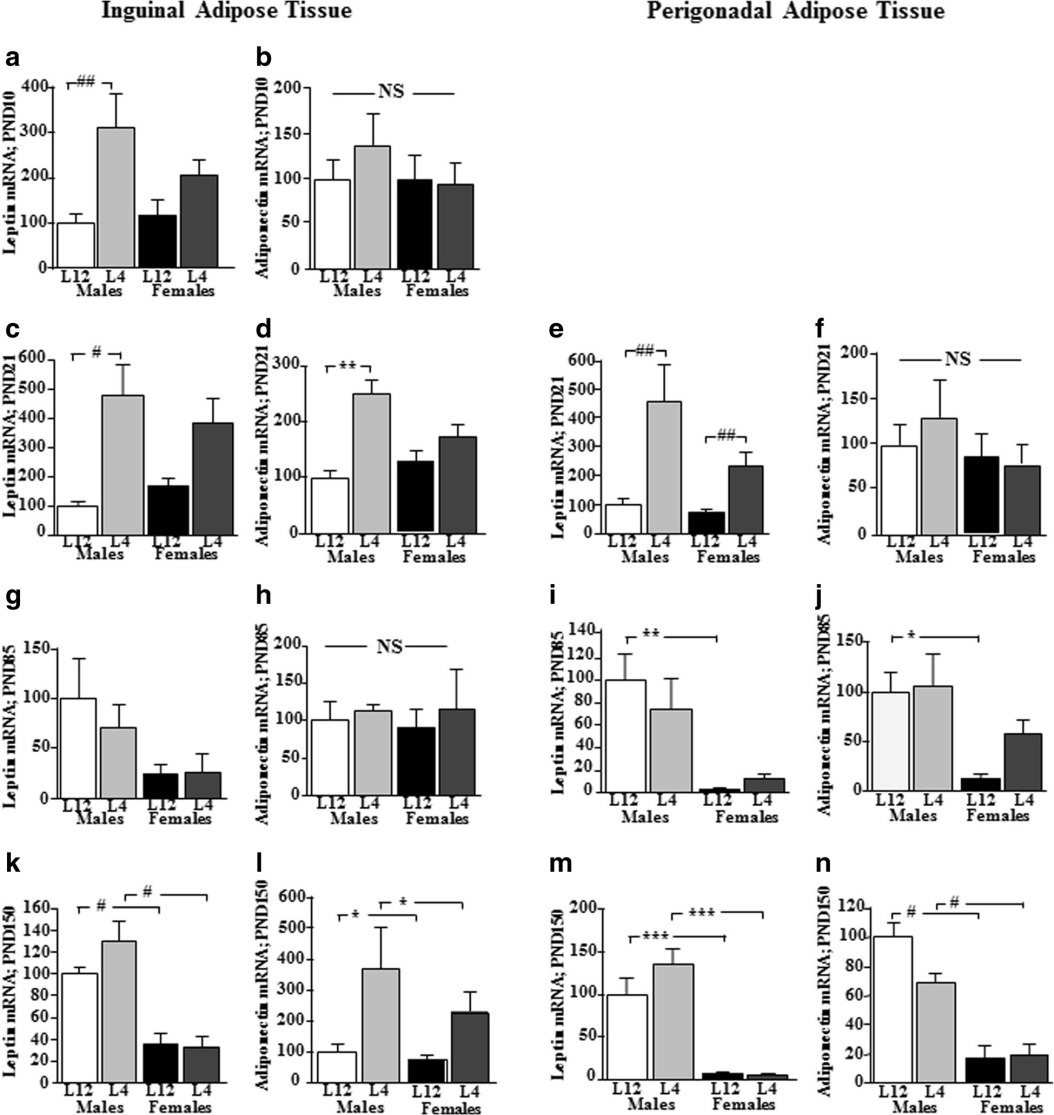
Mean mRNA levels of leptin and adiponectin in inguinal adipose tissue (*left column*) and perigonadal adipose tissue (*right column*) of male (M) and female (F) rats reared in litters of 4 (L4) or 12 (L12) pups at postnatal day (PND) 10 (**a**, **b**), PND 21 (**c**–**f**), PND85 (**g**–**j**), and PND150 (**k**–**n**). *NS* not statistically significant. #*p* < 0.0001, ##*p* < 0.002, ###*p* < 0.0002,*p < 0.01, **p < 0.001, ***p < 0.005. *N* = 6 in each experimental group at each age

At PND10, leptin mRNA levels were higher in L4 rats (litter effect: *F*_(1,16)_ = 12.9, *p* < 0.002; Fig. 3a), with this being statistically significant in males. IL1β mRNA levels were reduced in L4 females compared to those from L12 (litter effect: *F*_(1,16)_ = 11.6; *p* < 0.004; Table 3). There were no differences in adiponectin (Fig. 3b), IL6, or TNFα mRNA levels (Table 3).

**Table 3:** Relative mRNA levels of cytokines in inguinal and perigonadal adipose tissue at postnatal days (PND) 10, 21, 30, 50, 85, and 150 in male (M) and female (F) rats raised in litters (L) 4 or 12 pups

	Il1β mRNA inguinal	IL6 mRNA inguinal	TNFα mRNA inguinal	Il1β mRNA perigonadal	IL6 mRNA perigonadal	TNFα mRNA perigonadal
ML12PND10^d^	100.0 ± 21.3	100.0 ± 10.5	100.0 ± 10.5	TNA	TNA	TNA
ML4PND10^d^	100.5 ± 29.6	99.6 ± 56.6	88.9 ± 13.2	TNA	TNA	TNA
FL12PND10^d^	156.4 ± 44.6	94.9 ± 22.4	89.8 ± 12.3	TNA	TNA	TNA
FL4PND10^d^	32.4 ± 13.3^a^	38.7 ± 12.1	54.3 ± 11.1	TNA	TNA	TNA
ML12PND21^d^	100.0 ± 55.4	100.0 ± 38.7	100.0 ± 28.9	100.0 ± 23.7	100.0 ± 18.5	100.0 ± 21.5
ML4PND21^d^	42.5 ± 9.3	19.3 ± 1.7^a^	95.0 ± 25.7	70.3 ± 21.5	339.8 ± 73.8^a^	161.4 ± 53.6
FL12PND21^d^	97.8 ± 42.5	112.3 ± 46.6	142.1 ± 32.9	18.4 ± 4.7^b^	72.8 ± 20.1	50.2 ± 13.8
FL4PND21^d^	51.3 ± 23.6	20.7 ± 8.2^a^	41.7 ± 6.3	36.2 ± 8.1	331.0 ± 79.9^a^	75.4 ± 13.2
ML12PND85	BLD	BLD	BLD	100.0 ± 42.1	100.0 ± 28.9	100.0 ± 28.8
ML4PND85	BLD	BLD	BLD	70.0 ± 19.3	124.3 ± 30.3	124.3 ± 30.3
FL12PND85	BLD	BLD	BLD	47.7 ± 29.1	25.5 ± 20.1^b^	31.8 ± 24.7^b^
FL4PND85	BLD	BLD	BLD	51.0 ± 23.5	22.9 ± 12.4^b^	15.8 ± 9.1 ^b^
ML12PND150	BLD	BLD	BLD	100.0 ± 29.5	BLD	100.0 ± 39.5
ML4PND150	BLD	BLD	BLD	80.5 ± 37.0	BLD	450.5 ± 186.6 ^a^
FL12PND150	BLD	BLD	BLD	703.9 ± 142.6^b^	BLD	211.0 ± 49.4
FL4PND150	BLD	BLD	BLD	617.3 ± 145.8^b^	BLD	256.4 ± 65.2

*N* = 5–6 in each experimental group at each age

*TNA* tissue not available, *BLD* below limit of detection

^a^Different from rats of same age and sex (litter effect)

^b^Different from males of same age and litter size

^c^Different from preceding age (same sex and litter size)

^d^Non-fasting samples

At PND21, leptin mRNA levels were increased by litter size (*F*_(1,19)_ = 19.27, *p* < 0.0001), but this was only significant in males (*F*_(3,22)_ = 7.1, *p* < 0.002; Fig. 3c). There was an overall effect of litter size on adiponectin mRNA levels (*F*_(1,19)_ = 20.7, *p* < 0.0001), with an interaction between litter and sex (*F*_(1,19)_ = 5.73, *p* < 0.03) with L4 having higher levels than L12 in males (*F*_(3,22)_ = 8.72, *p* < 0.001; Fig. 3d). There was also a litter size effect on IL6 mRNA levels (*F*_(1,19)_ = 6.5, *p* < 0.02; Table 2) with L12 rats having higher levels than L4 rats in both sexes. This trend did not reach significance for IL1β or TNFα mRNA levels.

At PND85, leptin mRNA levels were sex dependent (*F*_(1,18)_ = 5.3, *p* < 0.04, Fig. 3g), with females having overall lower levels than males and this difference being statistically significant between rats from litters of 12. Adiponectin mRNA levels were also affected by sex (*F*_(1,24)_ = 18.1, *p* < 0.0001), with L12 males having higher levels than L12 females (Fig. 3h). IL1β, IL6, and TNFα mRNA (Table 2) levels were below the reliable detection levels of this assay in most samples.

At PND150, leptin mRNA levels were higher in males regardless of litter size (*F*_(1,18)_ = 62.8, *p* < 0.0001; Fig. 3k). There was a litter size effect on adiponectin mRNA levels (*F*_(1,19)_ = 7.1, *p* < 0.02), with rats of both sexes raised in small litters having higher adiponectin levels (Fig. 3l). Cytokine expression was below the reliable limits of detection of the assay.

#### Perigonadal adipose tissue

At PND10, there was insufficient perigonadal adipose tissue for analysis.

At PND21, the expression of leptin in perigonadal adipose tissue was affected by litter size (*F*_(1,20)_ = 5.3, *p* < 0.05; Fig. 3e), with L4 having higher levels than L12 in both sexes. There was no effect on adiponectin mRNA (Fig. 3f) levels. There was an effect of sex (*F*_(1,19)_ = 12.7, *p* < 0.002) on IL1β mRNA levels, with L12 males having higher levels than L12 females (Table 2). IL6 mRNA levels were higher in L4 pups compared to L12 pups of both sexes (*F*_(3,23)_ = 6.6, *p* < 0.01). TNFα mRNA levels were affected by sex (*F*_(1,20)_ = 4.9, *p* < 0.04), with males having overall higher levels than females (Table 2) but with no significant differences between groups in the post hoc test.

At PND85, there was an overall effect of sex on leptin mRNA levels (*F*_(1,18)_ = 18.8, *p* < 0.0001; Fig. 3i) and adiponectin (*F*_(1,24)_ = 18.1, *p* < 0.0001; Fig. 3j), with control males having higher mRNA levels of both adipokines than control females. There was an overall effect of sex on IL1β (*F*_(1,19)_ = 8.9, *p* < 0.008), IL6 (*F*_(1,18)_ = 23.9, *p* < 0.0001) and TNFα (*F*_(1,19)_ = 22.6, *p* < 0.0001) mRNA levels (Table 3), with IL6 (*F*_(3,18)_ = 5.1, *p* < 0.01) and TNFα mRNA levels being significantly different between males and females regardless of litter size (*F*_(3,22)_ = 7.6, *p* < 0.002). The differences between specific groups in IL1β mRNA levels did not reach significance in the post hoc test.

At PND150, leptin expression continued to be lower in females compared to males (*F*_(1,18)_ = 71.3, *p* < 0.0001; Fig. 3m) and adiponectin mRNA levels were also lower in females (*F*_(1,19)_ = 63.8, *p* < 0.0001; Fig. 3n), with the effect at this age being found regardless of litter size. IL1β mRNA levels were elevated in females compared to males (*F*_(1,19)_ = 29.4, *p* < 0.0001), regardless of litter size. TNFα mRNA levels were higher in males from L4 compared to those from L12 (litter size: *F*_(1,19)_ = 9.9, *p* < 0.005). IL6 mRNA levels were below the detection limit in most of the samples and therefore are not reported.

### Effect of neonatal testosterone treatment on metabolic parameters

#### PND10

**Fig. 4:**
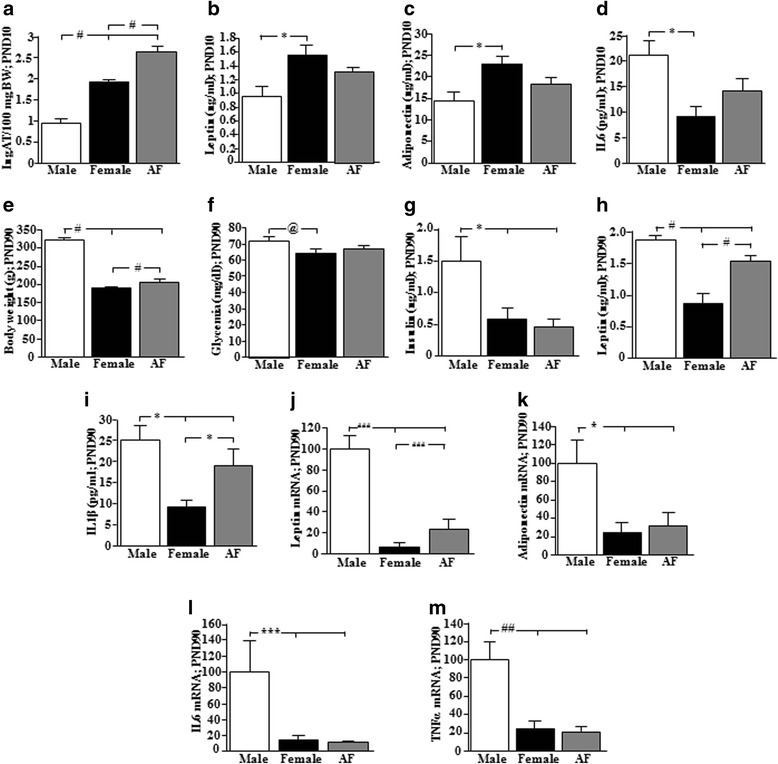
Metabolic changes in males, females, and females that were androgenized (AF) on postnatal day (PND) 1. At PND10, there were significant differences in **a** the percentage of inguinal adipose tissue (IngAT) and serum **b** leptin, **c** adiponectin, and **d** interleukin (IL) 6 levels. At PND90, there were significant differences in **e** body weight, **f** glycemia and serum, **g** insulin, **h** leptin and **i** IL1β levels. The mRNA levels of **j** leptin, **k** adiponectin, **l** IL6, and **m** TNFα in perigonadal adipose tissue at PND90 were also affected. #*p* < 0.0001, ##*p* < 0.002, ###*p* < 0.0002, *p < 0.01, ***p < 0.005, @*p* < 0.05. PND10: inguinal adipose tissue: *N* = 10; serum leptin and IL6 levels: *N* = 6; serum adiponectin: M and F: *N* = 8, AF: *N* = 9. PND90: body weight and glycemia: M: *N* = 8, F: *N* = 12, AF: *N* = 11; serum insulin, leptin, and Il1β levels: *N* = 6; mRNA levels for leptin, adiponectin, IL6, and TNFα: *N* = 6

At PND10, circulating testosterone levels remained elevated in females that received testosterone on PND1 (M: 6.3 ± 3.1, F: 1.1 ± 0.4, AF: 6.6 ± 2.5 ng/ml), with no difference in 17β-estradiol levels (M: 37.2 ± 4.0, F: 39.7 ± 6.1, AF: 28.4 ± 2.3 pg/ml). There was no effect of this treatment on body weight (M: 17.7 ± 0.4, F: 17.8 ± 0.4, AF: 18.3 ± 0.4 g) or length (M: 7.4 ± 0.1, F: 7.5 ± 0.1, AF: 7.3 ± 0.1 cm). However, the percentage of inguinal adipose tissue was increased (*F*_(2,27)_ = 55.6, *p* < 0.0001; Fig. 4a), with females having more than males and androgenized females more than both males and females.

Glycemia (M: 112.3 ± 3.1, F: 104.1 ± 4.0, AF: 102.1 ± 3.7 mg/dl) and insulin levels (M: 0.69 ± 0.24, F: 0.72 ± 0.19, AF: 0.49 ± 0.07 ng/ml) were unaffected at PND10. However, serum leptin levels were higher in females compared to males (*F*_(2,15)_ = 6.3, *p* < 0.01; Fig. 4b), but androgenized females were not different from either males or control females. Females also had higher circulating adiponectin levels than males (*F*_(2,22)_ = 5.6, *p* < 0.01; Fig. 4c), with neonatal androgenization reducing adiponectin levels, so that they were not different from males. There was no effect on circulating IL1β (M: 41.8 ± 24.7, F: 39.2 ± 9.2, AF: 27.1 ± 14.9 pg/ml) or TNFα levels (M: 1.7 ± 0.3, F: 1.1 ± 0.4, AF: 1.0 ± 0.4 pg/ml). In contrast, females had lower circulating IL6 than males, with androgenization of females ablating this difference (*F*_(2,15)_ = 6.0, *p* < 0.01; Fig. 4d).

In inguinal adipose tissue, leptin (M: 100 ± 26.3, F: 138.8 ± 21.7, AF: 142.9 ± 27.2 % M), adiponectin (M: 100 ± 21.0, F: 130.4 ± 9.5, AF: 103.7 ± 15.3 % M), IL1β (M: 100 ± 23.4, F: 59.0 ± 15.2, AF: 82.0 ± 14.7 % M), IL6 (M: 100 ± 20.8, F: 74.1 ± 16.3, AF: 94.1 ± 17.6 % M), and TNFα (M: 100 ± 13.2, F: 79.0 ± 9.1, AF: 97.9 ± 11.5 % M) mRNA levels did not differ between groups.

#### PND90

At PND90, circulating testosterone levels were no longer elevated in androgenized females, with males having higher levels than both female groups (M: 14.3 ± 3.7, F: 1.5 ± 0.4, AF: 1.2 ± 0.1 ng/ml; *F*_(2,26)_ = 13.5, *p* < 0.0001) and with no difference between groups in 17β-estradiol levels (M: 49.1 ± 2.1, F: 47.6 ± 2.8, AF: 42.1 ± 2.2 pg/ml).

Males weighed more than females and androgenized females, but androgenized females weighed more than control females (*F*_(2,28)_ = 128.6, *p* < 0.0001; Fig. 4e). The percentage of both inguinal adipose tissue (M: 0.55 ± 0.03, F: 0.42 ± 0.02, AF: 0.43 ± 0.02, *F*_(2,18)_ = 7.9, *p* < 0.004) and perigonadal adipose tissue (M: 1.46 ± 0.10, F: 0.94 ± 0.10, AF: 1.05 ± 0.08, *F*_(2,20)_ = 7.9, *p* < 0.004) was higher in males compared to both female groups, with no effect of neonatal testosterone treatment.

At PND90, glycemia was higher in males compared to control females and androgenization increased glucose levels, so that they were no longer different from males (*F*_(2,28)_ = 3.7, *p* < 0.05; Fig. 4f), but insulin levels were lower in both female groups (*F*_(2,15)_ = 5.1, *p* < 0.01; Fig. 4g).

Serum leptin levels were lower in females compared to males and neonatal testosterone treatment resulted in leptin levels being higher than in control females but still lower than in males (*F*_(2,15)_ = 22.4, *p* < 0.0001; Fig. 4h). Serum IL1β levels were lower in females compared to males and neonatal androgenization increased the levels of this cytokine in adult female rats (*F*_(2,14)_ = 5.6, *p* < 0.01; Fig. 4i). Serum adiponectin (M: 58.8 ± 6.2, F: 63.2 ± 4.7. AF: 63.9 ± 4.8 ng/ml) and IL6 (M: 10.6 ± 2.9, F: 18.6 ± 7.7, AF: 17.0 ± 2.6 pg/ml) were not different between groups. Circulating TNFα levels were below limit of detection.

The expression of leptin, adiponectin, and TNFα did not differ significantly between groups in inguinal adipose tissue (data not shown), while IL1β and IL6 were not reliably detectable in most samples.

In perigonadal adipose tissue, leptin mRNA levels were higher in males compared to females of both groups, but androgenization of females increased leptin expression so that it was higher than in normal females (*F*_(2,14)_ = 18.5, *p* < 0.0002; Fig. 4j). Adiponectin (*F*_(2,14)_ = 6.5, *p* < 0.01; Fig. 4k), IL6 (*F*_(2,13)_ = 9.7, *p* < 0.005; Fig. 4l), and TNFα (*F*_(2,14)_ = 11.7, *p* < 0.002; Fig. 4m) were lower in both groups of females compared to males. IL1β mRNA levels were not significantly different between groups (M: 100 ± 41.4, F: 30.6 ± 23.7, AF: 282.9 ± 154.9 % M).

## Discussion

The results presented here clearly demonstrate that age and sex must be taken into consideration when analyzing the effects of early overnutrition on body weight and diverse metabolic parameters. These results are also of importance for interpreting and reconciling conflicting outcomes of studies where the possible influence of litter size on metabolic parameters is not taken into account.

Here, we show that being raised in a small litter, which results in increased food intake during this early age due to greater maternal milk availability [25, 39], augmented body weight, adipose tissue, and serum glucose, insulin, leptin, and adiponectin levels during the perinatal period in rats of both sexes. Increases in body weight, adipose tissue mass, and insulin levels as a result of being raised in a small litter have been previously reported in nursing rats [28, 40, 41]. Here, we report that body length and adiponectin levels are also affected in both sexes at this early age.

The observed metabolic effects of neonatal overnutrition decreased or disappeared in adolescent and young adult rats, with some metabolic alterations reappearing in later adulthood. Litter size effects on body weight and leptin levels have been shown to vary with age in male rats, being present early in development and disappearing at 11 weeks of age [42], while Wiedmer and colleagues [43] reported that male rats from small litters weighed more than those from large litters from 2 to 5 weeks of age and then again from 10 to 12 weeks of age. Here, we show that these changes throughout development are different between males and females. For example, the effect of litter size on body weight was only found in males at the end of our study. Likewise, at the end of the study, the litter size effect on food intake was only observed in males. This is similar to what has been reported by Stefanidis and Spencer [28], who also suggested that there is a metabolic compensation by these rats in attempt to restore a normal body weight but that this compensation differs between males and females.

The early increase in body weight, length, and adipose tissue in L4 rats is most likely the direct result of an increase in nutritional intake during nursing [25, 44], while the long-term effects could be due to different factors that are modified by perinatal overfeeding. For example, changes in the early nutritional status modify neonatal leptin levels. Leptin is important for the development of neuronal circuits involved in metabolism [45–47] and changes in the levels of this hormone during critical periods of development can have long-term effects on metabolism [48]. Moreover, some of these affects do not become manifest until later in adulthood [19], as observed here. Leptin can also have direct effects on adipose tissue development [49]. Thus, as circulating leptin levels were increased at PND10 and PND21 in L4 rats of both sexes, changes in this hormone could play a role in the long-term effects of early overnutrition on metabolism.

Insulin also affects the development of metabolic neuronal circuits [50, 51] and in both males and females glycemia, and insulin levels were higher in neonatal L4 rats compared to L12 rats. Whether adiponectin modulates hypothalamic metabolic circuit development is unknown; however, levels of this adipokine in cord blood are positively correlated with leptin levels and adiposity in the newborn and adiponectin is suggested to play a role in early growth [52, 53]. Hence, the observed changes in metabolic hormones during neonatal overnutrition associated with the increase in weight and adipose tissue gain could influence the development of metabolic circuits and, thereby, condition metabolic status later in life.

Even after weaning when all rats had *ad libitum* access to food, L4 rats continued to eat more than L12 rats but only until approximately PND50. During adolescence and early adulthood, the effects of litter size on body weight and metabolic hormone levels also disappeared. Numerous changes occur during puberty that can affect metabolism. For example, the rise in sex steroids directly affects growth and fat accumulation [54, 55]. There are also modifications in the synthesis and release of neuropeptides involved in reproduction and growth [56–58], which could possibly affect hypothalamic metabolic neurocircuits. Thus, not only does early overnutrition affect the pubertal transition [23], but the pubertal transition also appears to modulate the metabolic response to early overnutrition. The intracellular signaling mechanisms involved in the adverse effects of aging on weight gain are similar to those activated in response to a poor diet [59]. Whether early overnutrition has an effect on metabolic aging, as possibly suggested by the reappearance of increased body weight and adipose tissue in later adulthood, deserves further attention.

Obesity has been associated with mild chronic systemic inflammation [29, 31, 60, 61]. We found no increase in circulating cytokines at any age in response to neonatal overnutrition. Moreover, in rats exposed to neonatal over nutrition, serum TNFα levels were actually decreased at PND10, with the expression levels of IL1β, IL6 and TNFα in inguinal adipose tissue tending to decrease in both sexes at PND10. Considering that these rats are still growing and adipocytes continue to proliferate, it is possible that the observed increase in adipose tissue could represent an increase in adipocyte number and not hypertrophy, as only the latter is associated with white adipose tissue inflammation. Moreover, as adipocytes differentiate, their expression of cytokines changes with, for example, only mature adipocytes producing leptin [62]. Hence, these early changes could also be representative of adipocyte proliferation and maturation in response to early overnutrition. This observation deserves further investigation. In contrast, at PND150, TNFα mRNA levels were significantly elevated in the perigonadal adipose of males that had been exposed to neonatal overnutrition. These male rats also had a higher body weight than their controls and this rise in cytokine production could represent the onset of peripheral inflammation, although circulating levels were unaffected.

At PND10, male rats from small litters had higher insulin levels, HOMA index, serum TNFα levels, and expression of IL1β in inguinal adipose but a lower percentage of inguinal adipose tissue than females from small litters. These early sex differences are clearly not due to the influence of postpubertal sex steroids, but we hypothesized that the neonatal testosterone surge that occurs in males [34] could be involved. Indeed, testosterone levels were increased at PND10 in L4 compared to L12 males, but the litter size had no effect on serum testosterone levels at any other age. Administration of testosterone to neonatal females has been classically used as an experimental model to study the mechanisms underlying sex differences in the brain [63]. This model has also been employed to analyze the long-term effects of increased neonatal androgen levels on the female reproductive axis and the development of polycystic ovary syndrome [64–67] and more recently on metabolic abnormalities [67–69]. Here, we found that testosterone levels remained increased at PND10 in females receiving a single injection of this sex steroid on PND1, but adult levels of testosterone and estradiol were unaffected. The higher levels of adiponectin in females at PND10 were partially masculinized in AF females and this could be due to the direct inhibitory effect of testosterone on adiponectin synthesis [70]. Likewise, the lower levels of IL6 in PND10 females were increased in androgenized females so that they were no longer different from males. This could be due to androgen effects on adipocyte differentiation, which can affect their expression of cytokines [71, 72]. However, neonatal androgenization exacerbated the sex difference in inguinal fat mass observed at PND10. Thus, some of the sex differences seen at PND10 and the different responses of males and females to being raised in a small litter could be at least partially due to an increase in testosterone levels observed in males.

There were long-term changes in metabolic parameters in response to neonatal androgenization, as previously reported [73, 74]. Although body weight and glycemia were slightly increased at PND90 in androgenized females, the most drastic effects were observed on circulating leptin and IL1β levels, which rose above normal female values. The rise in serum leptin correlated with increased expression in perigonadal adipose tissue, while the sex differences in perigonadal adipose expression of IL6 and TNFα were not affected by increased neonatal testosterone. Thus, the neonatal rise in testosterone in males could possibly play a role in the development of sex differences in adipose tissue function.

In the two studies reported here, we found some differences in the results of control males and females at PND10. For example, in the androgenization study PND10 females had higher serum leptin and adiponectin and lower IL6 levels than males, while these differences were not observed in the developmental study. In the first study, females had lower levels of IL6 than males, but this did not reach statistical significance, most likely due to the statistical methods that had to be applied to the developmental study that took into consideration the comparisons between many groups and factors. Indeed, if a Student’s *t* test is applied to the results at PND10, this sex differences is also significant in the first study. In the androgenization study, PND10 females had a higher percentage of inguinal adipose tissue compared to males of the same age. In the developmental study, there was a tendency for this to occur in the control pups (L12), but it was not significant. The significant change in the percentage of adipose tissue in females of the androgenization study could underlie their higher levels of circulating leptin and adiponectin. We cannot explain why these differences in females reached significance in the androgenization study and not the developmental study, although there are some plausible possibilities. There was a slight difference in litter sizes between these two studies, as we employed litters of 10 pups in the androgenization study to emulate a more normal litter size. However, another factor that must be taken into consideration is that all pups in the androgenization study were handled and injected with either saline or testosterone on PND1. Saline injection has not only been shown to modify leptin levels at this early age [75], but early stress can also affect the response to other hormonal changes [76] and it is possible that this early stress differentially affects males and females. This could also possibly explain the difference between males and females in serum adiponectin levels in the neonatal androgenization study. Likewise, in the analysis of the adult animals, the fact that not all females were in the same estrous cycle state (all control females were in diestrus in the androgenization study, while 80 % were in estrous and 20 % in diestrous in the developmental study), could also impact on the outcomes, although we found no effect of the estrous stage in the latter study. However, it is clear that under similar conditions, testosterone treatment of females modified some metabolic parameters at both PND10 and PND90.

We would like to emphasize that the majority of the results in control male and female rats were similar between the two studies, with some of the discrepancies resulting from the necessity to employ different statistical analyses due to the study designs. At PND10, there were no differences between control males and females in bodyweight, serum glucose, insulin, IL1β or TNFα levels, or mRNA levels of leptin, adiponectin, IL1β, IL6, or TNFα in inguinal adipose tissue in either study. At PND85/90, males weighed more, had more inguinal adipose tissue, and higher mRNA levels of leptin, adiponectin, IL6, and TNFα in perigonadal adipose tissue than control females. However, the above mentioned caveats must be taken into consideration when interpreting these data.

Here, we show that a reduction in litter size results in changes in circulating levels of testosterone, leptin, insulin, and adiponectin during the neonatal period when metabolic circuits are maturing [45], suggesting that these modifications could play a role in the long-term outcomes of this experimental model of early overnutrition. Moreover, from the results reported here it is clear that both sex and age must be taken into consideration when analyzing the effects of early nutritional manipulations. Although overnutrition due to large differences in litter size cannot be directly applied to humans, it is clear that poor maternal and early neonatal nutrition most likely increase the propensity to gain weight. Whether males and females are differentially affected in humans by these early nutritional changes deserves further attention. One must also take into consideration that in these studies we only analyzed two adipose depots and it is possible that other adipose tissues respond differently than the inguinal and perigonadal adipose depots and that these results may not be directly representative of what occurs in humans.

## Conclusions

Early overnutrition results in numerous changes in metabolic factors that could impact on the development of the hypothalamus and adipose tissue, which would in turn affect their metabolic function in later life. The metabolic response during neonatal overnutrition differs between males and females and this could, at least in part, explain the differences in long-term metabolic responses to these early manipulations.
